# A national survey of career development according to gender and subspecialties among cardiologists in Japan

**DOI:** 10.1371/journal.pone.0317029

**Published:** 2025-01-10

**Authors:** Mai Shimbo, Atsuko Nakayama, Noriko Fukue, Fumie Nishizaki, Chisa Matsumoto, Satsuki Noma, Satoko Ohno-Urabe, Chizuko A. Kamiya, Sachiko Kanki, Tomomi Ide, Hideo Izawa, Tatsunori Taniguchi, Yoshio Kobayashi

**Affiliations:** 1 Department of Cardiovascular Medicine, The University of Tokyo, Tokyo, Japan; 2 Department of Computational Diagnostic Radiology and Preventive Medicine, The University of Tokyo, Tokyo, Japan; 3 Department of Cardiovascular Medicine, Sakakibara Heart Institute, Tokyo, Japan; 4 Department of Cardiology, Yamaguchi Prefectural Grand Medical Center, Yamaguchi, Japan; 5 Department of Cardiology, Hirosaki University Graduate School of Medicine, Hirosaki, Japan; 6 Department of Cardiology, Tokyo Medical University Hospital, Tokyo, Japan; 7 Center for Health Surveillance & Preventive Medicine, Tokyo Medical University Hospital, Tokyo, Japan; 8 Department of Cardiovascular Medicine, Nippon Medical School, Japan; 9 Division of Cardiovascular Medicine, Department of Internal Medicine, Kurume University School of Medicine, Kurume, Japan; 10 Department of Obstetrics and Gynecology, National Cerebral and Cardiovascular Center, Osaka, Japan; 11 Department of Thoracic and Cardiovascular Surgery, Osaka Medical and Pharmaceutical University, Osaka, Japan; 12 Department of Cardiovascular Medicine, Graduate School of Medical Sciences, Kyushu University, Fukuoka, Japan; 13 Department of Cardiology, Fujita Health University, Aichi, Japan; 14 Department of Cardiovascular Medicine, Osaka University Graduate School of Medicine, Osaka, Japan; 15 Department of Cardiovascular Medicine, Chiba University Graduate School of Medicine, Chiba, Japan; Shiraz University of Medical Sciences, ISLAMIC REPUBLIC OF IRAN

## Abstract

**Background:**

Training opportunities, work satisfaction, and the factors that influence them according to gender and subspecialties are understudied among Japanese cardiologists.

**Methods:**

We investigated the career development of Japanese cardiologists with an e-mail questionnaire. Feelings of inequality in training opportunities, work dissatisfaction, and reasons were assessed by examining the cardiologists’ gender and invasiveness of subspecialties.

**Results:**

Responses were received from 2,566 cardiologists. Female cardiologists were underrepresented in invasive subspecialties compared to males (14.2% vs. 85.8%, p<0.0001). In both invasive and non-invasive subspecialties, female cardiologists felt more inequality in training opportunities than males (invasive: 50.0% vs. 36.2%, non-invasive: 41.6% vs. 30.9%, p<0.001, respectively) and were less satisfied with their work (invasive: 26.0% vs. 18.3%, non-invasive: 24.7% vs. 14.7%, p = 0.001, respectively). Although female cardiologists in invasive subspecialties did not feel significantly more inequal and dissatisfied than those in non-invasive subspecialties (p = 0.063 and p = 0.758, respectively), male cardiologists in invasive subspecialties felt more inequal and dissatisfied than those in non-invasive subspecialties (p = 0.015 and p = 0.040, respectively). Female cardiologists were more influenced by gender bias and family issues for inequality in training opportunities (p = 0.0001, respectively), whereas male cardiologists were likely to be affected by specifications of belonging hospitals. Both genders felt dissatisfied when their expectations were unmet and they were overworked.

**Conclusions:**

Female cardiologists felt more inequality regarding training opportunities and dissatisfaction with career development than male cardiologists in both the invasive and non-invasive subspecialties. Diversity support is warranted for achieving satisfying career course regardless of gender and subspecialty.

## Introduction

Globally, the number of female physicians has increased. In Japan, 22.8% of physicians were female in 2020 [1], and the proportion of females is higher in the younger generations. We previously reported that female and younger cardiologists experience more inequality regarding training opportunities and lower work satisfaction [2]. Moreover, male cardiologists perceived less inequality in training opportunities as female cardiologists increased, indicating that workplace diversity may positively impact both female and male cardiologists. To establish a better work environment, knowing the reasons for feelings of inequality in training opportunities and dissatisfaction with work is important; however, there is insufficient research on them.

In cardiology, subspecialties vary widely; for example, imaging, heart failure, or cardiac rehabilitation are non-invasive subspecialties, and intervention, arrhythmia, or surgery are invasive subspecialties. Subspecialty significantly impacts the lifestyle and career development of cardiologists. Currently, females are underrepresented in cardiology worldwide with the data of around 10–20% of cardiologists consisted of female, particularly in invasive subspecialties with approximately 4–5% of female cardiologists in interventional subspecialty [3, 4]. This situation is similar to Japan [2, 5]. Yong et al. reported that fewer female cardiologists were interested in intervention subspecialties compared to males, and the factors for females, such as having an interest in subspecialties other than intervention, little flexibility in work opportunities over a lifetime, gender discrimination or harassment, were negatively influenced by this unbalanced situation [6]. These factors may continuously impact cardiologists’ career development and influence feelings of inequality in training opportunities or work dissatisfaction in both female and male cardiologists. However, these have not been well studied among Japanese cardiologists after choosing a subspecialty, especially among cardiologists by subspecialty and gender.

This study aimed to investigate the career development of cardiologists and perceptions of equality in training opportunities and work satisfaction according to cardiologists’ subspecialty and gender, using a qualitative survey method to identify the background factors that may obstruct career development with the aim of improving the workplace environment.

## Methods

### Study design

This is a sub-analysis of our previous survey conducted by our research team from the Committee for Diversity Promotion of the Japanese Circulation Society (JCS) as the cross-sectional survey conducted with a 31-item questionnaire to investigate cardiologists’ career development and satisfaction with their work. The questionnaire was well reviewed by the members of the Committee for Diversity Promotion of the Japanese Circulation Society, which made up of university professors and cardiovascular experts. Career development was defined as acquiring expertise and working as a specialist through experience and progress with advancement opportunities as a cardiologist. As other research have done [6, 7], a 31-item questionnaire including open section for comments was designed to obtain demographic information, such as gender, age, subspecialties in cardiology, family care status, and belonging hospital, and if they feel inequality or work dissatisfaction or not were collected to examine differences in career development according to gender and subspecialty. The questionnaire was reviewed by the members of the Committee for Diversity Promotion of the JCS. The details of the survey questionnaire have been provided previously [2].

A Google form was used for the questionnaire, and valid responses included those who answered all the questions in the survey. The questionnaire was sent by e-mail to 14,798 cardiologists who were JCS members in September 2022. The survey was open from September 13 to 30, 2022. Survey responses were obtained from 2,566 cardiologists (17.3% response rate), and all personal information was removed.

The study was conducted under the ethical principles of the Declaration of Helsinki. This study was approved by the JCS Ethics Committee (ID:15). Before they answered the questionnaire, JCS members were announced that they would be regarded as consenting to our study protocol approved by the JCS Ethics Committee by completing the questionnaire.

### Outcomes

Feelings of inequality in training opportunities and overall dissatisfaction with work for career development among cardiologists were assessed according to gender and subspecialty. The term ‘inequality’ represents the differences of chances in training opportunity during the career course as a cardiologist compared to colleges including experiencing of gender harassment and family circumstances. The term ‘work satisfaction’ represents overall satisfaction including feelings of happiness through his/her work as a cardiologist, pay, promotions, supervision, working environment and family circumstances. Feeling more inequality in training opportunities and work dissatisfaction were recognized from the sum of ‘Yes’ and ‘Often Yes’ for the feeling of inequality in training opportunities and work dissatisfaction questions. These feelings were compared between the female and male cardiologists. Subspecialties were divided into two groups. Invasive treatment subspecialties included ischemic heart disease, arrhythmia including catheter ablation, vascular and cardiac surgery (adult and pediatric), structural heart disease, and emergency medicine. Non-invasive treatment included heart failure, echocardiography and diagnostic imaging, pediatric cardiovascular and pediatric cardiovascular congenital heart disease, pulmonary hypertension, cardiac rehabilitation, hypertension and vascular disease, medical check-up, basic medicine, social medicine (public health) and others. We investigated the reasons for feelings of inequality in training opportunities and work dissatisfaction with free-text descriptions by respondents or by choosing categories.

### Statistical analysis

JCS member characteristics were compared using the χ2-test for non-continuous variables and an unpaired *t*-test for normative continuous data using SPSS v22 (IBM Inc., Armonk, NY, United States). Non-continuous variables are presented as percentages and normally distributed continuous data are presented as the mean ± SD. A logistic regression analysis was used to assess the effect of each reason for work dissatisfaction. The significance level was set at an alpha value of 0.05.

## Results

### Background of respondents

Respondents’ characteristics are presented in Table 1. The ratio of female cardiologists in this study was almost the same as the ratio of female physicians in Japan. Of the 2,566 valid responses, female cardiologists in invasive subspecialties (n = 184, 7.1%) were 44.7 ± 8.7 years old, with 49.5% being of younger age (<45 years), while male cardiologists in the same fields (n = 1110, 43.3%) were 49.0 ± 9.9 years old with 35.7% being of younger age (<45 years). In invasive subspecialties, the rate of female cardiologists was lower than those in non-invasive fields (14.2 vs. 34.6%, p<0.0001). Female cardiologists in invasive subspecialties had fewer family care duties compared to female cardiologists in non-invasive subspecialties (50.5 vs. 63.6%, p = 0.002), and male cardiologists in invasive subspecialties (50.5 vs. 70.7%, p = 0.0001).

**Table 1:** Background of participated cardiologists.

	Invasive subspecialties				p-value	Non-invasive subspecialties				p-value
	Female		Male			Female		Male		
**Total numbers, n (%)**	184	(14.2)	1110	(85.8)	0.0001	440	(34.6)	832	(65.4)	0.0001
**Age, years ± SD**	44.7 ± 8.7		49.0 ± 9.9		0.0001	45.9 ± 9.8		51.4 ± 11.4		0.0001
<45 years old, n (%)	90	(49.5)	391	(35.7)		200	(46.9)	254	(30.9)	
45 years old≦, n (%)	92	(50.5)	705	(64.3)		226	(53.1)	567	(63.6)	
**Years of experience, n (%)**					0.001					0.001
<3	5	(2.7)	8	(0.7)		23	(5.2)	25	(3.0)	
3–5	8	(4.3)	30	(2.7)		25	(5.7)	24	(2.9)	
6–10	31	(16.8)	110	(9.9)		58	(13.2)	79	(9.5)	
11–20	81	(44.0)	383	(34.5)		179	(40.7)	225	(27.0)	
≧21	59	(32.1)	579	(52.2)		155	(35.2)	479	(57.6)	
**Having a doctor degree, n (%)**					0.002					0.001
Yes	129	(70.1)	895	(80.6)		336	(76.4)	732	(88.0)	
No	55	(29.9)	215	(19.4)		104	(23.6)	100	(12.0)	
**Working hospital, n (%)**					0.550					0.011
University hospital	58	(31.5)	346	(31.2)		168	(38.2)	318	(38.2)	
City hospital (<10 doctors)	48	(26.1)	299	(26.9)		100	(22.7)	129	(15.5)	
City hospital (10–19 doctors)	30	(16.3)	135	(12.2)		32	(7.3)	73	(8.8)	
City hospital (20 doctors <)	34	(18.5)	215	(19.4)		63	(14.3)	130	(15.6)	
Clinic	11	(6.0)	104	(9.4)		62	(14.1)	161	(19.4)	
Others	3	(1.6)	11	(1.1)		15	(3.4)	21	(2.5)	
**Need of family caring, n (%)**					0.0001					0.985
Yes	93	(50.5)	785	(70.7)		280	(63.6)	529	(63.6)	
No	91	(49.5)	325	(29.3)		160	(36.4)	303	(36.4)	
**Sub-specialty, n (%)**										
**Invasive**					0.178					
Ischemic heart disease	81	(44.0)	572	(51.5)			-		-	
Arrythmia	73	(39.7)	304	(27.4)			-		-	
Vascular and cardiac surgery	15	(8.2)	120	(10.8)			-		-	
Structural heart disease	9	(4.9)	51	(4.6)			-		-	
Emergency medicine	6	(3.3)	63	(5.7)			-		-	
**Non-invasive**										0.004
Echocardiography & diagnostic Imaging		-		-		156	(35.5)	132	(15.9)	
Heart failure		-		-		71	(16.1)	257	(30.9)	
Medical checkup		-		-		40	(9.1)	39	(4.7)	
Cardiac rehabilitation		-		-		36	(8.2)	49	(5.9)	
Basic medicine		-		-		27	(6.1)	73	(8.8)	
Hypertension/Vascular disease		-		-		24	(5.5)	112	(13.5)	
Pediatrics/Congenital heart disease		-		-		20	(4.5)	65	(7.8)	
Pulmonary hypertension		-		-		7	(1.6)	23	(2.8)	
Others		-		-		28	(6.4)	50	(6.0)	
None		-		-		21	(4.8)	26	(3.1)	
Considering		-		-		10	(2.2)	6	(0.7)	

P-values were obtained by a chi-squared test comparing female and male cardiologists by subspecialties.

In invasive subspecialties, female cardiologists were underrepresented compared to male cardiologists (14.2 vs. 85.8%, p = 0.0001). Meanwhile, the rate of female cardiologists was higher than that of male cardiologists in non-invasive subspecialties, including imaging, medical checkups, cardiac rehabilitation, and basic science.

### Inequality in training opportunities and overall satisfaction

**Fig 1:**
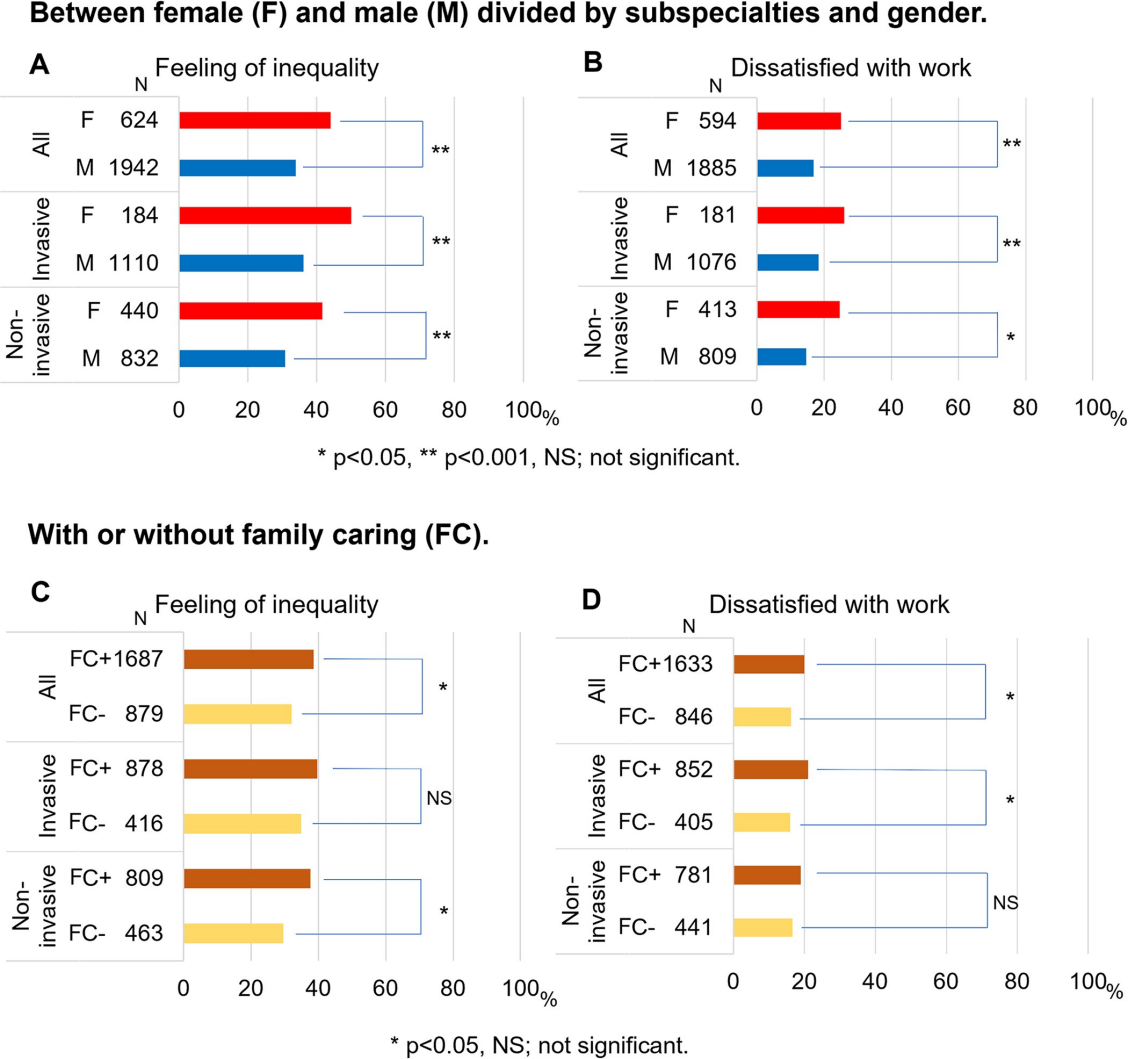
The rate of feeling of inequality in training opportunities and dissatisfaction with work among cardiologists. A, B. Inequality in training opportunities and work dissatisfaction between female and male divided by subspecialties. C, D. Inequality in training opportunities and work dissatisfaction among cardiologists with or without family care. (FC: family care; F: female; I: invasive; M: male; NI: non-invasive).

Regarding training opportunities, female cardiologists perceived more inequality in training opportunities than males in both invasive and non-invasive subspecialties (invasive: 50.0% vs. 36.2%, p = 0.0001; non-invasive: 41.6% vs. 30.9%, p = 0.0001, respectively) (Fig 1A). Females were also less satisfied with their work than males in both invasive and non-invasive subspecialties (invasive: 26.0% vs. 18.3%, p = 0.019; non-invasive: 24.7% vs. 14.7%, p = 0.0001, respectively) (Fig 1B).

Between invasive or non-invasive subspecialties divided by gender, both female and male cardiologists in invasive subspecialties tended to feel more inequality in training opportunities than those in non-invasive subspecialties (50.0% vs. 41.6%, p = 0.06; 36.2% vs. 30.9%, p = 0.014, respectively). Male invasive cardiologists were less satisfied than those in non-invasive subspecialties (18.3% vs. 14.7%, p = 0.040). Although female cardiologists were less satisfied than male cardiologists, there were no significant differences in work dissatisfaction between invasive and non-invasive subspecialties among female cardiologists (26.0% vs. 24.7%, p = 0.758).

Cardiologists with family care duties in non-invasive subspecialties felt more inequality in training opportunities than those without such duties (37.5% vs. 29.6%, p = 0.005), while cardiologists in invasive subspecialties did not (39.7% vs. 34.9%, p = 0.098) (Fig 1C).

In contrast, in the invasive subspecialties, cardiologists with family care duties were less satisfied with their work than cardiologists without such duties (21.0% vs. 16.0%, p = 0.039), whereas cardiologists in the non-invasive subspecialties were not (19.0% vs. 16.6%, p = 0.315) (Fig 1D).

### Reasons for feeling of inequality in training opportunities

**Fig 2:**
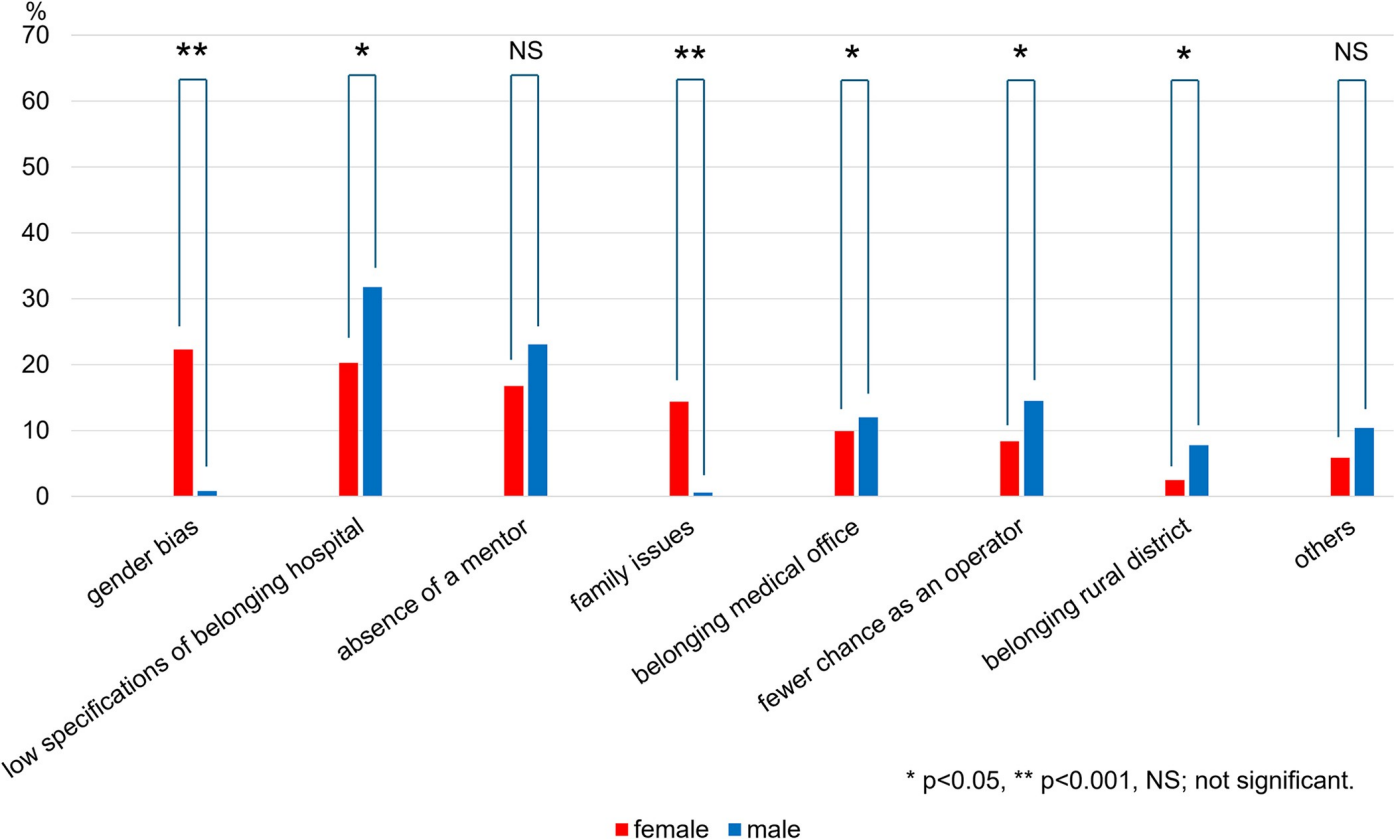
Reasons for feeling of inequality in training divided by gender. The rate of cardiologists in each reason for feeling of inequality in training opportunities in female and male cardiologists divided by subspecialties.

Among the respondents who chose ‘Yes’ and ‘Often Yes’ for the feeling of inequality in training opportunities, the following reasons for inequality in training opportunities with free-text responses were extracted: 1) gender bias and discrimination, 2) low specifications of belonging hospitals such as can’t perform urgent catheterization, 3) absence of a mentor, 4) family issues, 5) belonging medical office, in which an organizational academic system used in departments of university hospitals, is not good, 6) fewer chance as an operator, and 7) belonging rural district, 8) others (Fig 2). When disaggregated by subspecialty, female cardiologists were more affected by gender bias and family issues than male cardiologists, regardless of whether they were invasive (affected by gender bias; 23.2% vs. 0.3%, p = 0.0001; affected by family issues: 8.7% vs. 0.6%, p = 0.001, respectively) or non-invasive (affected by gender bias; 21.8% vs. 1.5%, p<0.0001; affected by family issues: 17.3% vs. 0.5%, p = 0.0001, respectively) (Table 2). On the other hand, male cardiologists in invasive subspecialties were influenced more by the low specification of belonging hospitals than females (33.2% vs. 17.4%, p = 0.009), while male cardiologists in non-invasive subspecialties were influenced more by the absence of a mentor than females (26.8% vs. 17.3%, p = 0.046).

**Table 2:** Reasons for feeling of inequality in training opportunities by subspecialties.

	Invasive subspecialties				p-value	Non-invasive subspecialties				p-value
	Female		Male			Female		Male		
Gender bias, n (%)	16	(23.2)	1	(0.3)	0.0001	29	(21.8)	3	(1.5)	0.0001
Low specifications of belonging hospital, n (%)	12	(17.4)	105	(33.2)	0.009	29	(21.8)	57	(29.4)	0.159
Absence of a mentor, n (%)	11	(15.9)	66	(20.9)	0.409	23	(17.3)	52	(26.8)	0.046
Family issues, n (%)	6	(8.7)	2	(0.6)	0.001	23	(17.3)	1	(0.5)	0.0001
Belonging medical office, n (%)	10	(14.5)	42	(13.3)	0.846	10	(7.5)	19	(9.8)	0.555
Fewer chance as an operator, n (%)	9	(13.0)	50	(15.8)	0.712	8	(6.0)	24	(12.4)	0.061
Belonging rural district, n (%)	2	(1.5)	14	(7.2)	0.019	3	(4.3)	26	(8.2)	0.325
Others, n (%)	7	(5.3)	22	(11.3)	0.650	5	(7.2)	22	(11.3)	0.074

P-values were obtained by a chi-squared test comparing female and male cardiologists by subspecialties.

### Reasons for work dissatisfaction

**Fig 3:**
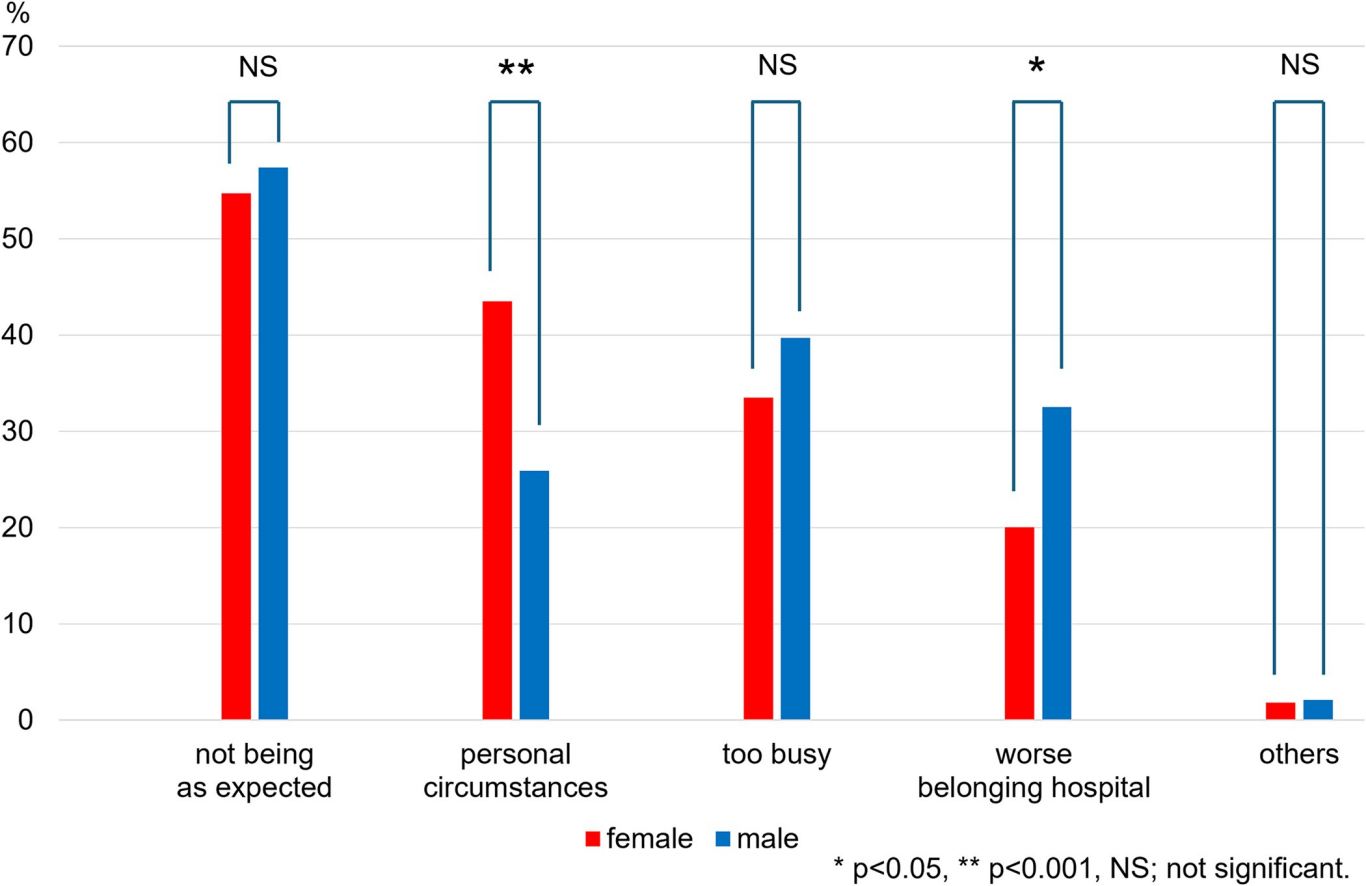
Reasons for work dissatisfaction divided by gender. The rate of cardiologists in each reason for work dissatisfaction in female and male cardiologists divided by subspecialties.

The questions with prepared choices to search for reasons for dissatisfaction with their work included 1) not being as expected, 2) personal circumstances, 3) too busy, 4) worse belonging hospital, and 6) others (Fig 3). Regardless of gender or invasive or non-invasive subspecialties, cardiologists were less satisfied if they were not being as they expected (54.7% females vs. 57.4% males, p = 0.56) (Fig 3) or too busy (33.5% females vs. 39.7% males, p = 0.17) (Fig 3). Female cardiologists were less satisfied with their personal circumstances than male cardiologists in invasive and non-invasive subspecialties (invasive: 51.0% vs. 24.3%, p = 0.0001; non-invasive: 40.3% vs. 28.4%, p = 0.040, respectively) (Table 3).

**Table 3:** Reasons for work dissatisfaction divided by subspecialties.

	Invasive subspecialties				p-value	Non-invasive subspecialties				p-value
	Female		Male			Female		Male		
Not being as expected, n (%)	27	(52.9)	132	(57.4)	0.747	66	(55.5)	85	(57.4)	0.562
Personal circumstances, n (%)	26	(51.0)	56	(24.3)	0.0001	48	(40.3)	42	(28.4)	0.040
Too busy, n (%)	14	(27.4)	82	(35.7)	0.264	43	(36.1)	68	(45.9)	0.106
Worse belonging hospital/office, n (%)	11	(21.6)	74	(32.2)	0.136	23	(19.3)	49	(33.1)	0.012

P-values were obtained by a chi-squared test comparing female and male cardiologists by subspecialties.

A logistic regression analysis of reasons for work dissatisfaction revealed that ‘not being as expected’ was significantly correlated with dissatisfaction in invasive (Odds ratio (OR) 3.90; 95% confidence interval (CI) 1.79–8.47; p = 0.001) and non-invasive subspecialties (OR 5.82; 95% CI 2.71–12.5; p = 0.001) (Table 4).

**Table 4:** Logistic regression analysis for dissatisfaction.

	Invasive subspecialties			Non-invasive subspecialties		
	OR	95% CI	p-value	OR	95% CI	p-value
**Reasons for dissatisfaction**						
Not being as expected	3.90	1.79–8.47	0.001	5.82	2.71–12.5	0.001
Personal circumstances	1.52	0.73–3.19	0.267	0.97	0.47–1.91	0.878
Too busy	0.44	0.21–0.90	0.024	0.33	0.17–0.66	0.002
Worse belonging hospital/office	2.33	0.93–5.84	0.072	2.57	1.03–6.39	0.043

(CI, confidence interval; OR, odds ratio.)

## Discussion

In our nationwide questionnaire survey, female cardiologists in invasive and non-invasive subspecialties felt more inequality in training opportunities than male cardiologists and were less satisfied with their work than male cardiologists. On the other hand, male cardiologists in the invasive subspecialty felt more inequality in training opportunities and were less satisfied with work than those in the non-invasive subspecialty, while female cardiologists had no significant difference between invasive and non-invasive subspecialties. The reasons for feeling inequality in training opportunities differed by gender, with female cardiologists listing more gender bias and family issues than male cardiologists. Cardiologists not being as they expected was the most frequent reason for dissatisfaction, regardless of gender and subspecialty.

### Feeling inequality of training opportunities and dissatisfaction with work by gender and subspecialties

Previous reports, including ours, have indicated that female cardiologists feel more inequality in training opportunities and are less satisfied with their work than male cardiologists [2]. There have been reports of high inequity and difficult situations among females in invasive specialty or academia [8]. Lack of opportunity, radiation exposure, and a male-dominant culture without female role models and mentorship are reported to be barriers to female cardiologists achieving their careers as specialists in invasive subspecialties [9]. Unexpected working hours, on-call requirements, and hard physical demands are also considerable barriers for female cardiologists; however, Burgess et al. discussed that these are not definitive elements but significant problems with their workplace culture [4]. In this study, we revealed that female cardiologists in non-invasive subspecialties also felt more inequality and were less satisfied than male cardiologists in the same subspecialty. Joshi et al. reported that more than half of female cardiologists in cardiovascular imaging, which is divided into non-invasive subspecialties, faced unconscious or conscious bias, verbal harassment, burnout, and imposter syndrome at work [10]. Moreover, female cardiologists may face childbearing and subsequent maternity leave and play a major role in childcare and housework. These conditions are one of the reasons that females likely to take part-time work, which might lead to the situation such as difference in salary and job activities according to gender [11]. Raber et al. reported that investment in resources to support life events may be recommended to recruit and retain cardiologists dedicated to becoming parents and developing productive careers [12]. In Japan, females traditionally have more housework and family care responsibilities, which may limit their career development [7]. Our data demonstrated that fewer female cardiologists had family care duties than male cardiologists with invasive subspecialties, suggesting that female cardiologists with invasive subspecialties have a higher risk of having children with little work flexibility. It is necessary to change the consciousness of both female and male cardiologists regarding work-life balance and child-rearing through career development.

In comparison, divided by gender, male cardiologists in invasive subspecialties felt more inequality in training opportunities and were less satisfied with work than those in non-invasive subspecialties. Irregular working hours, on-call duties, and unfair case distribution may influence these situations. In contrast, although female cardiologists underrepresented operators of structural heart procedures or were frequently unmarried and childless in invasive subspecialties [13, 14], female cardiologists showed no significant differences between invasive and non-invasive subspecialties in feelings of inequality in training opportunities and work dissatisfaction in this study. This may be due to the statistical matters with the limited number of female cardiologists in invasive subspecialties. However, unconscious gender bias and imposter syndrome may lead females to feel ‘Females are like this after all, I gave up’ in male-dominated cultures [4, 10]. In addition, it is reported that female academic physicians have lower salary than male physicians, and one of the possible explanations are receiving less recognition for achievements and overt discrimination [15]. Such situation is also seen in Japan. Japanese females were traditionally expected to be quiet and unobtrusive lady and ‘walk three steps behind male’. In fact, Holman et al reported that gender gap was especially pronounced in Japan, and Japan had fewer female authors of academic papers among research-producing countries [16]. Although this atmosphere is improving in the younger generation, females are still required more effort than males in male-dominant culture. It is difficult to change long-standing culture, however, pioneer cardiologists have visualized the real-world situation, extracted problems and published and announced to the world [7]. This suggests that female cardiologists should be aware of the need to establish unshakable positions in their workplaces. Indeed, female and male cardiologists should consider workplace diversity such as establishing communication platform, adopting successful diversity systems and give equal training opportunities and mentoring to one who wants to grow as a cardiologist, and a concept that satisfies both gender and subspecialty requirements is required.

### The reasons for inequality in training opportunities and work dissatisfaction by gender and subspecialties

Few studies have investigated the inequality in training opportunities and work dissatisfaction after cardiologists select their subspecialty. Our study revealed that female cardiologists were significantly influenced by gender bias and personal circumstances, including family issues, in both invasive and non-invasive subspecialties for feeling of inequality in training opportunities compared to male cardiologists, whereas male cardiologists were more likely to be influenced by belonging hospital or medical office than females.

Notably, the number of male cardiologists who experienced gender bias or family issues as a burden was extremely low compared to female cardiologists. Supportive role models and positive encouragement have been reported as the most popular factors leading trainees to subspecialty selections [17]. In fact, there are gender differences in the reasons for choosing subspecialties at the beginning of career development. In the intervention field, female cardiologists tend to choose an interventional subspecialty when they have a female mentor or role model, whereas male cardiologists are more likely to be influenced by being an expert or the possibility of employment after training completion [6]. Numerous studies have shown that females are more prone to face unconscious bias, discrimination, or harassment than males in each situation, subspecialty, specialty, and country [2, 4, 10, 17], even though there is no evidence that females are inferior to male [18, 19]. Various efforts, such as continuous mentoring, support for female faculty development programs, and an environment of flexible work styles, are progressing to overcome these unbalanced situations. As our results demonstrated, reasons for feeling inequality in training opportunities differ by gender, suggesting that female cardiologists need support for their personal or social environment, such as childcare support systems or having a mentor who can protect them from discrimination regardless of subspecialty, while male cardiologists may need a platform that allows them to find a well-equipped hospital or medical office suitable for their career planning.

Other interesting findings of this study were that the most popular reason for less work satisfaction, ‘being not as expected,’ was common in both genders and subspecialties. It is important to pursue career development as one wants and to be excited about coming to work each day for lifelong work satisfaction [20]. Kohno et al. reported that satisfaction with cardiovascular training among early career cardiologists was associated with the volume of invasive procedures or the opportunity to experience novel therapeutic measures [21]. However, these factors are probably less likely to influence mid-to-veteran-career cardiologists. Overwork was another popular reason for less work satisfaction. Flexibility of working hours is reported to be one of the key points for female cardiologists [22], as well as for male cardiologists. Taken together, achieving one’s ideal career course and work-life balance are vital and universal desires for cardiologists, regardless of gender and subspecialty. Diverse workplaces may reduce gaps between the ideal and real-world situations. Continuous evaluation of cardiologists’ current situation, sharing and picking up opinions how they want to be, and awareness of their wishes for career development are warranted.

### Limitations

The response rate of our survey was relatively low. The answers to the questionnaire may indicate more interest in a cardiologist’s career development than non-answerers, which may differ from the usual population within a circulating society, and cause the reporter bias. Moreover, the ratio of female responders (24.3%) in this survey was relatively higher than the ratio of female cardiologists in Japan (13.5%) [2]. One of the possible reasons why the response rate of female cardiologists was higher than the ratio of female cardiologists in real-world situation is that female cardiologists may feel more inequality and dissatisfaction for their work and feel the need to appeal their current situation. The awareness of inequality in training opportunities and work dissatisfaction in career development and family care duties was self-reported and the feeling of the cardiologists, which was difficult to quantify. It did not indicate the actual inequality in the working environment and might be affected by gender bias. A prospective study of cardiologists’ desired plans and career development in cardiology is warranted to investigate the real-world environment.

## Conclusions

Female cardiologists felt more inequality in training opportunities and dissatisfaction in career development than male cardiologists in both the invasive and non-invasive subspecialties. Being as expected during career development and work-life balance are important for both genders. Diversity support is warranted for achieving satisfying career course regardless of gender and subspecialty.
